# Supplementary material for: Size and body condition drive the energetic cost of a baleen whale foraging in shallow habitat
Source: PeerJ. 2025 Oct 30;13:e20247. doi: 10.7717/peerj.20247 (PMC12579852; doi:10.7717/peerj.20247)
Supplement: Supplemental Information 1 [file peerj-13-20247-s001.pdf]

Supplementary Materials

Table of Contents

Ethogram ... ***2***

Drone Details ... ***4***

Nares Area ... ***5***

Snapshot selection criteria ... 5

Measuring ... 6

Sample Sizes ... 6

Left and right nares comparison ... 6

Total length (TL) and nares area ... 7

Blow Type Comparison ... 7

Metric Correlations ... ***9***

Respiration Models ... ***10***

Recovery Models ... 11

Anticipation Models ... 15

Recovery and Anticipation Model ... 18

Travel Model ... 18

Exploratory Plots ... 20

References ... ***21***

Ethogram

Table S1. Complete ethogram of all behaviours used to annotate footage. Taken from supplementary materials for Bird et al. (2024).

Primary behaviour state	Sub-behaviour tactics	Point or State	Definition
Foraging			
	Headstand	State	Whale is positioned head down-flukes up, or if in water depths less than whale body length, whale may be more horizontal in water column; With both body positions the whale is observed pushing head/mouth region into substrate.
	Side-swim (stationary)	State	Whale observed swimming on its side, but not moving forward. Characterised by frequent jaw snapping.
	Side-swim (forward)	State	Whale observed swimming on its side, moving forward. Characterised by frequent jaw snapping.
	Upside-down swim (forward)	State	Whale observed swimming upside-down, moving forward. Characterised by frequent jaw snapping.
	Subsurface (forward)	State	Whale swims subsurface while feeding. Characterised by frequent jaw snapping.
	Subsurface (stationary)	State	Whale maintains a stationary position while feeding below the surface of the water oriented dorsal up. Characterised by frequent jaw snapping.
	Surface feeding	State	Whale feeding right at the surface, frequently breaking the surface but without breathing. Characterized by frequent turning and frequent jaw snapping/flexing.
	Skim feeding	State	Whale swims at the surface with mouth open for an extended period. Characterised by moving forward in a straight line.
	Bubble blast	Point	Underwater release of air by whale that rises to surface and forms a circle/puka.
	Bubbles from mouth	Point	A stream of bubbles is observed emanating from the whale's mouth while it is at the surface.
	Open and closes mouth under water	Point	Mouth opens and closes in quick succession, like taking a bite.
	Open mouth at surface	State	The mouth of the whale is observed at the surface (so that the baleen is visible).
	Sediment from mouth	Point	A stream of sediment is observed emanating from the whale's mouth.

	Sediment from unknown source	Point	Sediment observed coming from unknown source, whale is headstanding but mouth not visible.
	Sharking	State	Whale's fluke observed above the water surface.
Social			Whales interacting with each other, usually involves some form of tactile interaction.
	Bump-tactile interaction	Point	Two adult whales are observed making body contact.
	Pair coordinated surfacings	State	Two whales surface together in close proximity multiple times (not a mother-calf pair).
	Promiscuous behaviour	State	A whale positions itself upside-down underneath the ventral side of another whale.
	Echelon swimming	State	Mother and calf observed swimming very closely together.
	Mother-calf tactile interaction	State	Mother and calf whales are observed touching each other through any body part (head, pectoral fins, body).
	Presumed Nursing	State	Calf is observed going into a nursing position under its mother's ventral surface and positioning there for an extended period.
	Pass under	State	Calf observed crossing under mother briefly so clearly not nursing.
	Pass over	State	Calf observed crossing over mother.
Rest			Logging type behaviour observed where whale remains in same location, lying at or just below the surface, and with minimal to no active fluking to promote movement. Surfacings are generally slow and at regular intervals.
Travel			Whale shows directed travel in a consistent direction, with regular surfacing intervals.
Other			
	Body Roll	Point	Whale observed moving its body in a barrel roll.
	Defecate	Point	Faeces is observed streaming from posterior end of whale (care is taken to avoid confusion with sediment emanating from mouth.
	Lower jaw flex	Point	Lower jaw of whale is quickly pushed/flared outward.
	Pec flare	Point	The whale swings its pectoral fin outward.
	Turning	State	Whale makes rapid change in direction (over a spatial scale of <20 m).
	Blow	Point	Whale exhales and inhales.
	Breach	Point	Whale is observed breaching out of the water.

Dive	Point	Whale dives to begin a long breath hold duration.
Fluke swish	Point	Rapid "swish" movement of fluke horizontally from side to side.
Swimming at surface	State	Whale is swimming slowly at the surface performed short breath hold dives in-between blows.
Sculling	State	Whale observed rotating pectoral fins in circles.
Spyhopping	State	Whale observed lifting head vertically out of the water.
Swerve	Point	Whale observed tilting/partially rolling to the side but not completely.

A video compilation of the foraging tactics is available here:
https://figshare.com/s/0a486c768e55ba0f7ea5

Drone Details

We used several drone models for data collection between 2016-2022 (Table S2). Each drone was piloted using manual remote flight control and real-time camera output through a tablet ground station. Each camera was stabilized by a 3-axis brushless gimbal. Video output was recorded at 4K and a 1080 p down sample was transmitted real-time to the pilot at 30 Hz. While each drone was equipped with a barometer for measuring altitude, the DJI Inspire 2 also contained a LiDAR altimeter (e.g., "LidarBoX", Bierlich et al., 2024) (Table S2). Taken from supplementary materials for Bird et al. (2024).

Table S2. Camera specifications associated with each unoccupied aircraft system (UAS) used in this study. Each UAS had a barometer to record the altitude of the drone during video collection, while the Inspire 2 also had an altimeter (LiDAR) (Bierlich et al., 2024; Dawson et al., 2017)

UAS	Years used	Sensor (mm)	Pixel resolution (pox)	Focal length lens (mm)	Altimeter
Phantom 3 Pro	2016, 2017	6.16 x 4.6	3840 x 2160	3.61	Barometer
Phantom 4	2016, 2017	6.16 x 4.6	3840 x 2160	3.61	Barometer
Phantom 4 Pro	2017, 2018, 2019	13.2 x 8.8	3840 x 2160	8.8	Barometer
Inspire 2	2020, 2021, 2022	17.3 x 13	3840 x 2160	25	Barometer & LiDAR

Nares Area

Snapshot selection criteria

Snapshot image quality was ranked following the criteria in Table S3. Only snapshots in category Y were measured for this analysis (Fig S1).

Table S3. Snapshot quality score ranking system.

Y	Yes/good quality/ measurable
O	Obstructed by blow
B	Blurry image quality
C	Closed nares
N/A	Nares not in shot

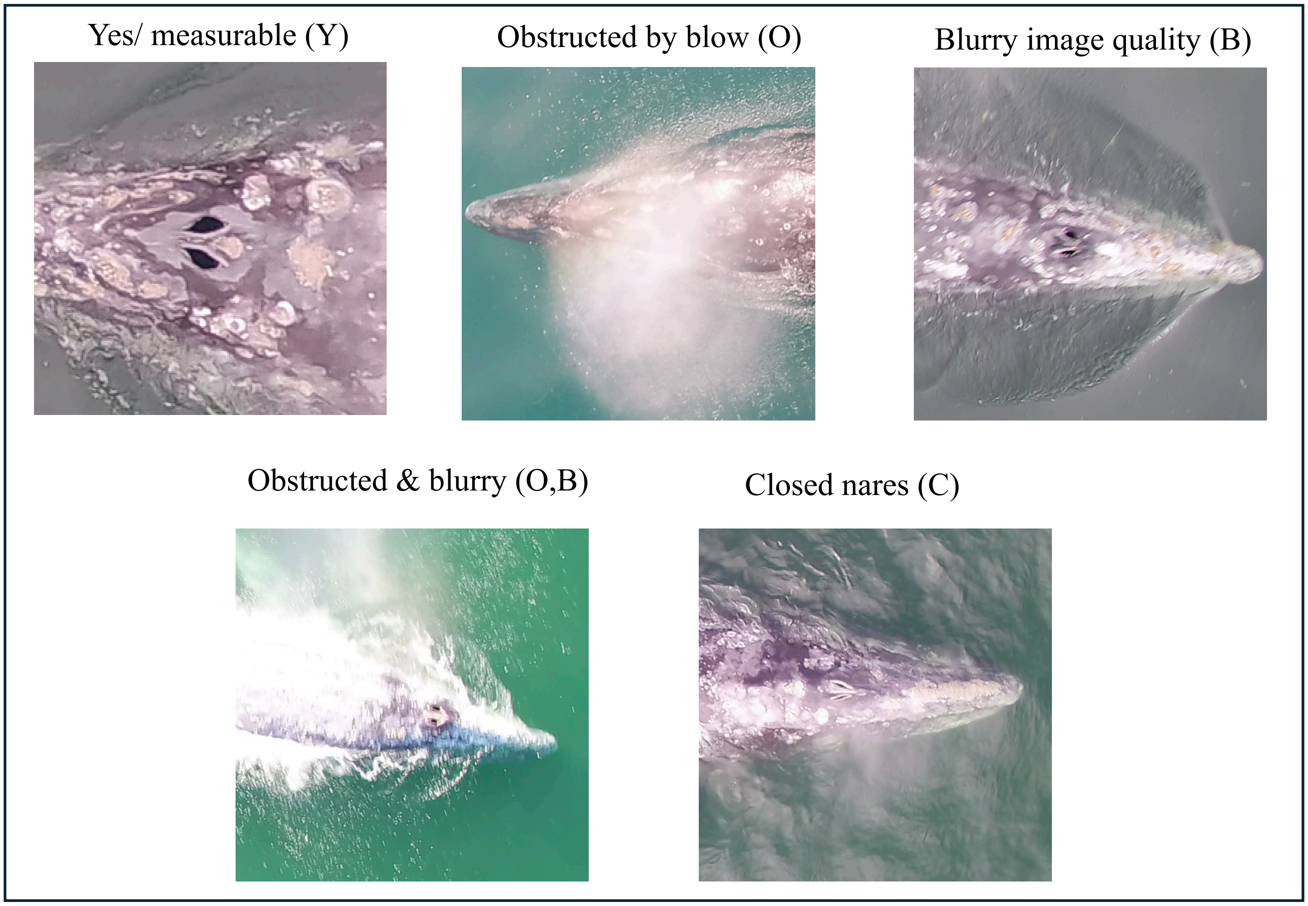

Figure S1. Examples of ranked snapshots.

Measuring

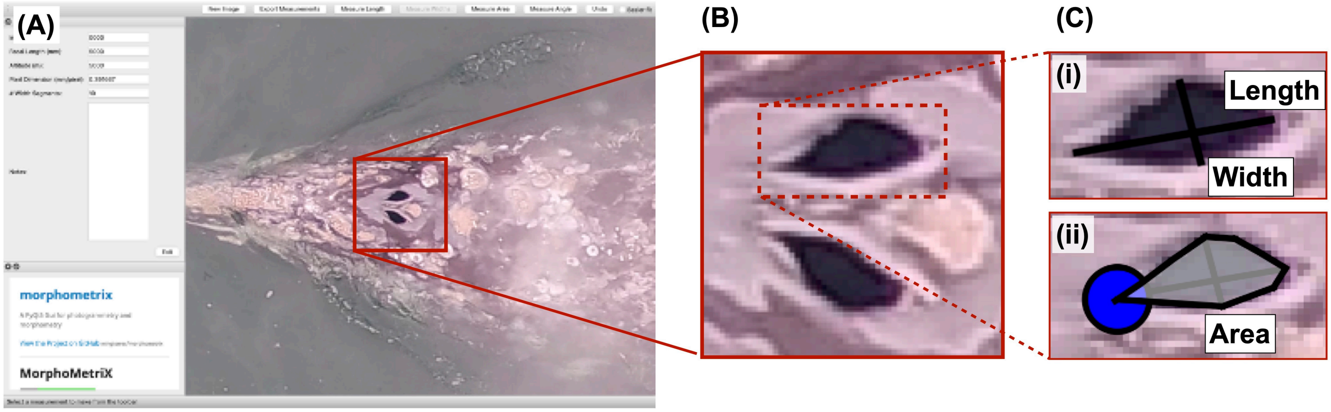

Figure S2. Measuring a naris in MorphoMetriX. (A) An image of the whale in MorphoMetriX, (B) Selecting which naris to measure, (C) measuring the (i) length and width, and (ii) area.

Sample Sizes

Table S4. A summary of the data used in this study, including the number of individual whales, sequences, and blows observed in each gross behavior state. The number of blows per sequence, inhalation duration, and naris area are also summarized by the mean and standard deviation.

Behavior State	No. Individuals	No. sequences	No. blows *(initial,middle,terminal)*	No. blows per sequence *mean (s.d)*	Naris area (m^2) *mean (s.d)*
**forage**	12	42	99 (18,39,42)	4 (3)	0.0019 (7e-04)
**travel**	15	28	84 (11,45,28)	6 (5)	0.0018 (0.00075)

Left and right nares comparison

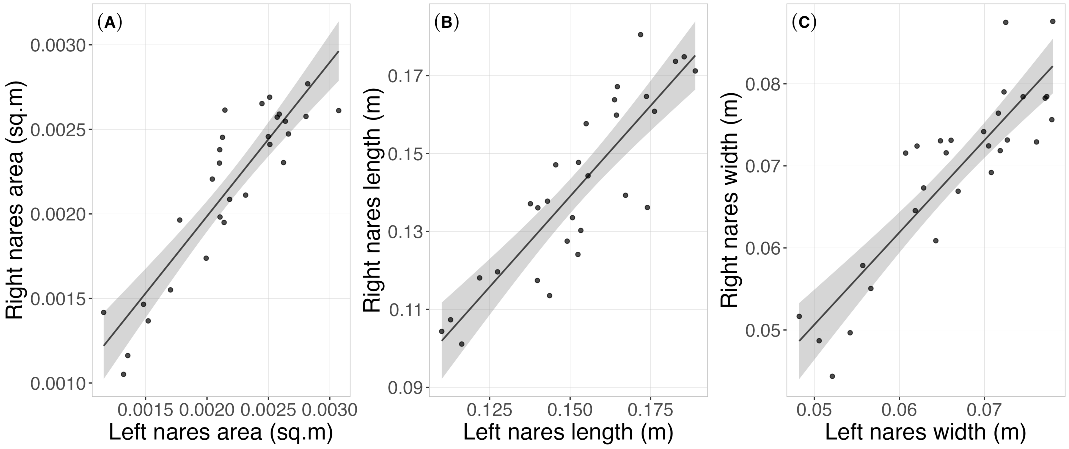

Figure S3. Relationships between (A) area, (B) length, and (C) width of the left and right nares. Gray shading represents the 95% confidence interval.

Total length (TL) and nares area

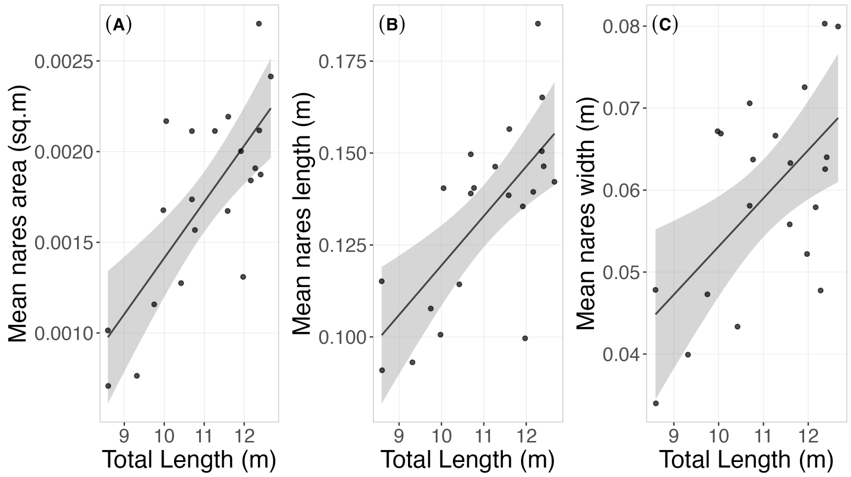

Figure S4. Relationships between total length (TL) and (A) mean nares area, (B) mean length, (C) mean width, and (D) inhalation duration. Gray shading represents the 95% confidence interval.

Blow Type Comparison

Table S5. Mean and standard deviation of naris area, and total length standardized nares area per blow type.

Blow Type	No. blows	Naris area (m^2) mean (s.d)	Standardized naris area mean (s.d)
initial	18	0.002 (0.00053)	1.5e-05 (3.2e-06)
middle	39	0.0021 (0.00063)	1.4e-05 (4.4e-06)
terminal	42	0.0017 (0.00078)	1.4e-05 (3.9e-06)

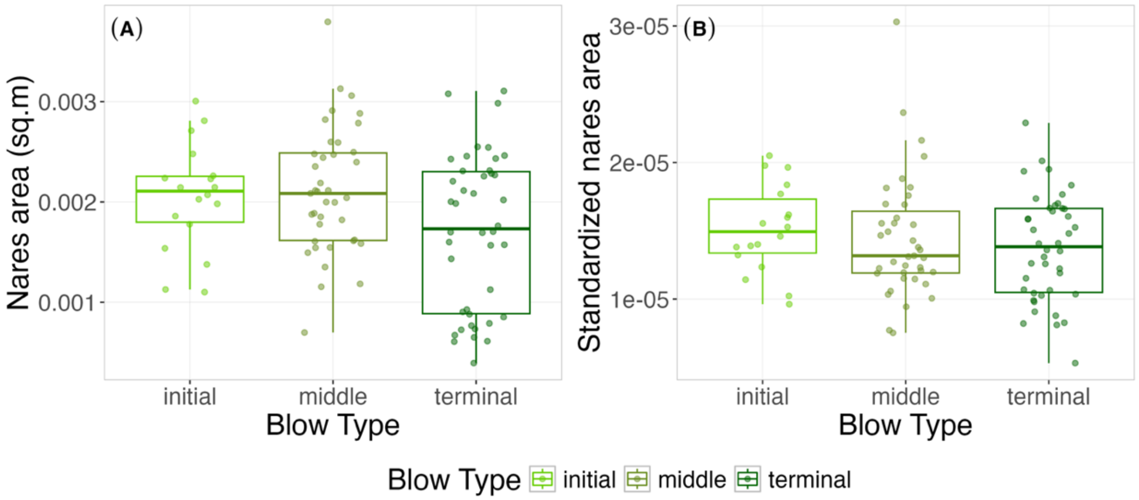

Figure S5. Variation in (A) absolute nares area (sq. m) and (B) nares area standardized by total length. Points are colored by blow type.

Metric Correlations

Table S6. Summaries of respiration metrics.

Behavior State	Metric	Mean (s.d)
Forage	Total inhalation duration (s)	3.22 (2.54)
	Interbreath interval (IBI;s)	23.51 (17.92)
	Rate of inhalation accumulation	0.09 (0.05)
	Initial breath duration (s)	1.38 (0.22)
	Terminal breath duration (s)	1.43 (0.28)
	Breath count (drone)	2.34 (1.95)
	Breath count (tag)	3.42 (2.54)
	Breath hold duration (s)	40.69 (33.38)
Travel	Respiration rate (drone; breaths min^{-1})	1.59 (1.01)
	Respiration rate (tag; breaths min^{-1})	0.43 (0.30)

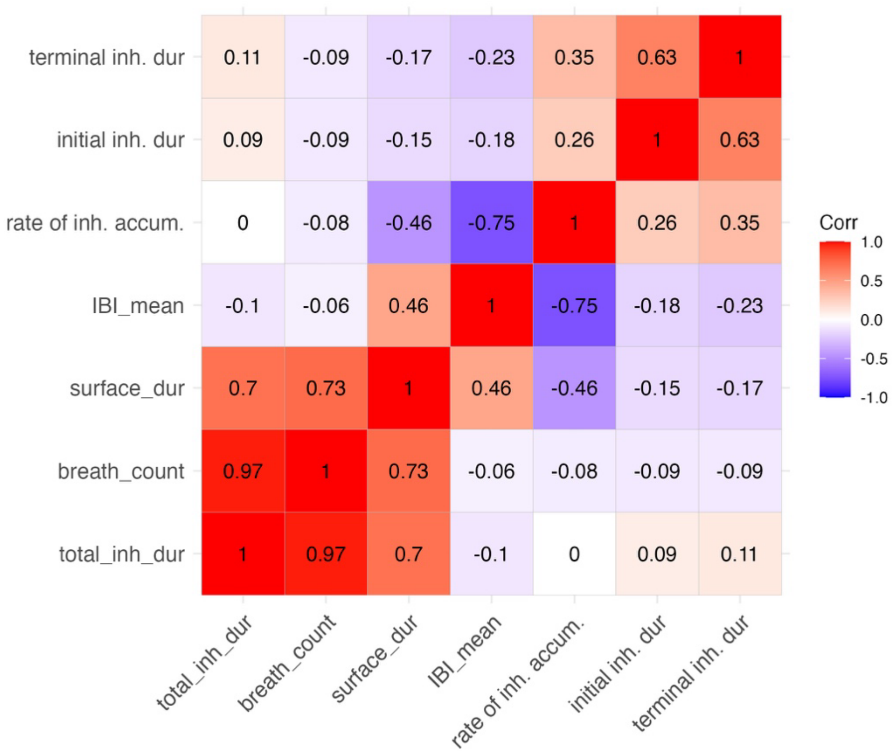

Figure S6. Correlation matrix for all respiration metrics considered for analysis.

Respiration Models

Table S7. Summary of sample sizes per model.

	Model	No. Seq	No. Ind	No. sequences per tactic					
				Headstand	***Side-swim (stationary)***	***Fwd. swim tactics***	***Sub surface stationary***	***Surface tactics***	***Benthic dig***
**Recovery**	Total Inhalation Duration	221	55	109	21	65	9	17	-
	Inter-breath Interval	143	48	87	8	38	4	6	-
	Inhalation accumulation rate	113	41	65	6	35	3	4	-
	Initial breath inhalation duration	228	56	116	21	65	9	17	-
	Breath count (Drone)	254	57	127	21	71	12	23	-
	Breath count (Tag)	667	9	135	-	180	-	-	352
**Anticipation**	Total Inhalation Duration	189	49	93	15	58	8	15	-
	Inter-breath Interval	118	43	67	11	35	1	4	-
	Inhalation accumulation rate	88	36	50	8	26	1	3	-
	Terminal breath inhalation duration	124	44	72	12	35	2	3	-
**Both**	Total Inhalation Duration	123	41	64	5	36	3	15	-
**Travel**	Respiration rate (Drone)	40	29	-	-	-	-	-	-

<u>*Recovery Models*</u>

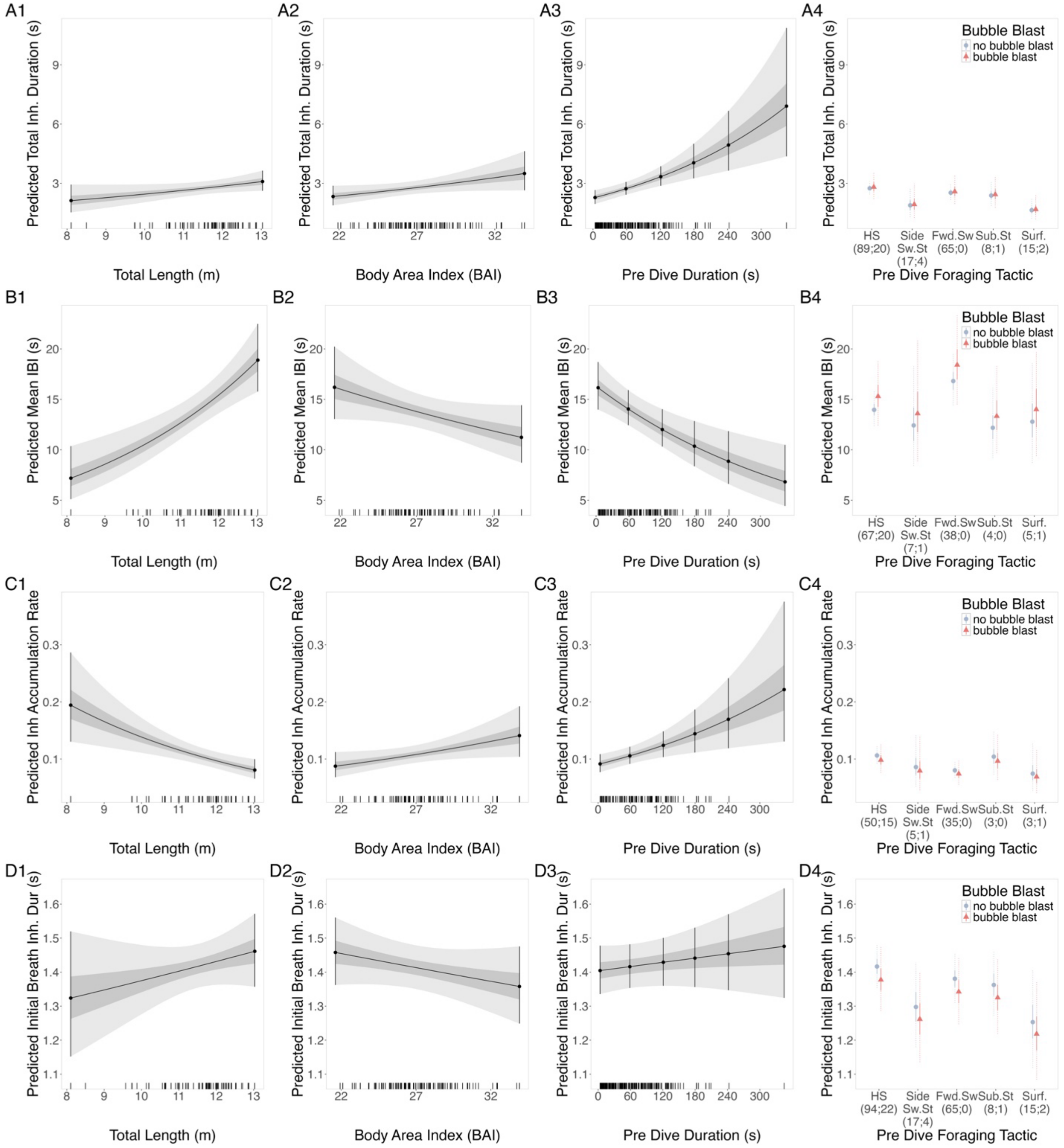

Figure S7. Estimated relationships from the "recovery models" between (1) total length (TL), (2) Body Area Index (BAI), (3) preceding dive duration (s) and (4) preceding dive foraging tactic and bubble blast occurrence and (A) total inhalation duration (s), (B) mean inter-breath interval (IBI), (C) inhalation accumulation rate, and (D) inhalation duration of the initial breath. All response variables were transformed from the log scale to seconds. In columns 1, 2, and 3, the line represents the mean posterior relationship, the dark gray shaded region represents the 50% credible interval, and the light gray shaded region represents the 95% credible interval. Points with bars represent the mean probability and 95% credible interval at the minimum and

maximum (1) TL and (2) BAI. In (3) preceding dive duration, points with bars represent the mean value and 95% credible interval at the minimum and maximum dive duration and 1-,2-,3-,4- minute dive durations. The rug of vertical black lines along the x-axis in each plot represents the original data values. In column 4, the points represent the posterior mean values, the solid lines represent the 50% credible intervals, and the dashed lines represent the 95% credible intervals. In column 4 the tactics have been abbreviated as follows: HS = Headstand, Side.Sw.St = Side-swim stationary, Fwd.Sw. = Forward swimming tactics, Sub.St = Subsurface stationary, Surf. = Surface tactics. Grey circles indicate that no bubble blast occurred, while orange triangles indicated the occurrence of a bubble blast. The sample sizes per tactic are reported in parentheses under the tactic name: the first value indicates the number of observations with no bubble blast for that tactic, and the second value indicates the number of observations with a bubble blast for that tactic. The y-axis is fixed within each response variable.

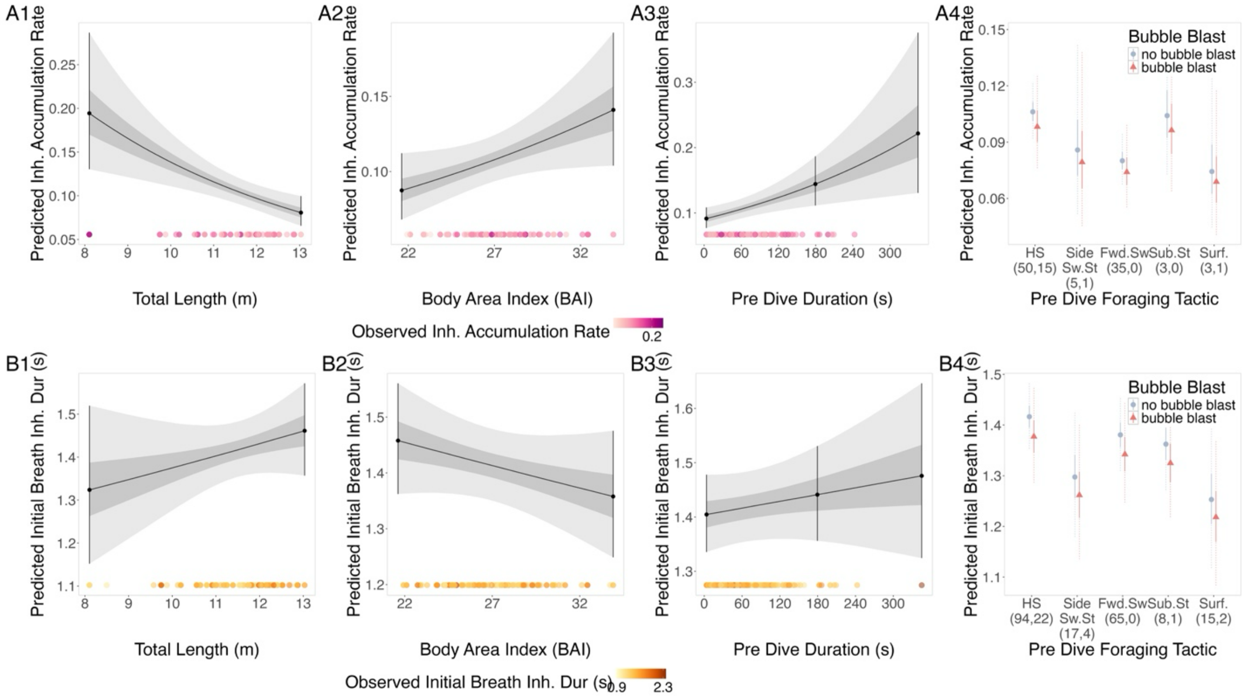

Figure S8. Estimated relationships from the "recovery models" between (1) total length (TL), (2) Body Area Index (BAI), (3) preceding dive duration (s) and (4) preceding dive foraging tactic and bubble blast occurrence (A) inhalation accumulation rate, and (B) inhalation duration of the initial breath, All response variables were transformed from the log scale to seconds. In columns 1, 2, and 3, the line represents the mean posterior relationship, the dark gray shaded region represents the 50% credible interval, and the light gray shaded region represents the 95% credible interval. Points with bars represent the mean probability and 95% credible interval at the minimum and maximum (1) TL and (2) BAI. In (3) preceding dive duration, points with bars represent the mean value and 95% credible interval at the minimum and maximum dive duration and 3-minute dive durations. The points along the x-axis in each plot represents the original data values, colored by the observed values of each row's respective response variable with darker shades representing higher values. In column 4, the points represent the posterior mean values, the solid lines represent the 50% credible intervals, and the dashed lines represent the 95%

credible intervals. In column 4 the tactics have been abbreviated as follows: HS = Headstand, Side.Sw.St = Side-swim stationary, Fwd.Sw. = Forward swimming tactics, Sub.St = Subsurface stationary, Surf. = Surface tactics. Grey circles indicate that no bubble blast occurred, while orange triangles indicated the occurrence of a bubble blast. The sample sizes per tactic are reported in parentheses under the tactic name: the first value indicates the number of observations with no bubble blast for that tactic, and the second value indicates the number of observations with a bubble blast for that tactic.

Table S8. Model coefficients for all four recovery models. All dive variables pertain to the dive preceding the surface series.

			**Total Inhalation Duration**	**Inter-breath Interval (IBI)**	**Inhalation accumulation slope**	**Initial breath inhalation duration**
**name**	**coefficient**	**prior**	**mean (s.d)**	**mean (s.d)**	**mean (s.d)**	**mean (s.d)**
Intercept	a	normal(0,1)	1.01 (0.89, 1.13)	2.65 (0.06)	-2.24 (0.07)	0.35 (0.02)
TL	bT	normal(0,1)	0.08 (0.05)	0.21 (0.05)	-0.19 (0.06)	0.02 (0.02)
BAI	bBAI	normal(0,1)	0.08 (0.04)	-0.07 (0.04)	0.09 (0.05)	-0.01 (0.01)
Bubble blast occurrence	bBB_pre	normal(0,1)	0.02 (0.13)	0.09 (0.1)	-0.08 (0.13)	-0.03 (0.03)
Preceding dive duration	bDD_pre	normal(0,1)	0.16 (0.04)	-0.13 (0.04)	0.13 (0.04)	0.01 (0.01)
Forward swim tactics	bSWF_pre	normal(0,1)	-0.09 (0.1)	0.18 (0.1)	-0.28 (0.11)	-0.03 (0.03)
Subsurface stationary	bSBS_pre	normal(0,1)	-0.38 (0.19)	-0.13 (0.21)	-0.21 (0.27)	-0.09 (0.05)
Side-swim stationary	bSSS_pre	normal(0,1)	-0.14 (0.13)	-0.15 (0.15)	-0.02 (0.19)	-0.04 (0.03)
Surface/skim feeding	bSUF_pre	normal(0,1)	-0.51 (0.16)	-0.1 (0.21)	-0.35 (0.27)	-0.12 (0.06)
Individual level random effect	sigma_id	uniform(0,10)	0.09 (0.06)	0.22 (0.06)	0.2 (0.08)	0.13 (0.02)

Table S9. Posterior predictive check results for all four recovery models.

**Model**	**Posterior p-value for mean(y)**	**Posterior p-value for s.d(y)**
Total Inhalation Duration	0.490	0.681
Inter-breath Interval (IBI)	0.479	0.675
Inhalation accumulation slope	0.523	0.705
Initial breath inhalation duration	0.499	0.618

Table S10. Model coefficients for the two recovery models comparing UAS data to tag data. All dive variables pertain to the dive preceding the surface series.

			**Breath Count (UAS)**	**Breath Count (tag)**
**name**	**coeff**	**prior**	**mean (s.d)**	**mean (s.d)**
Intercept	a	normal(0,1)	0.83 (0.07)	1.05 (0.17)
TL	bT	normal(0,1)	0.07 (0.05)	0.08 (0.1)
BAI	bBAI	normal(0,1)	0.11 (0.05)	0.14 (0.14)
Bubble blast occurrence	bBB_pre	normal(0,1)	-0.02 (0.13)	-
Preceding dive duration	bDD_pre	normal(0,1)	0.16 (0.04)	0.2 (0.02)
Forward swim tactics	bSWF_pre	normal(0,1)	-0.15 (0.12)	-
Side-swim forward	bSSF_pre	normal(0,1)	-	0.17 (0.08)
Subsurface stationary	bSBS_pre	normal(0,1)	-0.39 (0.25)	-
Side-swim stationary	bSSS_pre	normal(0,1)	-0.02 (0.16)	-
Surface tactics	bSUF_pre	normal(0,1)	-0.44 (0.21)	-
Benthic dig	bBEN_pre	normal(0,1)	-	0 (0.06)
Individual level random effect	sigma_id	uniform(0,10)	0.08 (0.06)	0.39 (0.19)

<u>*Anticipation Models*</u>

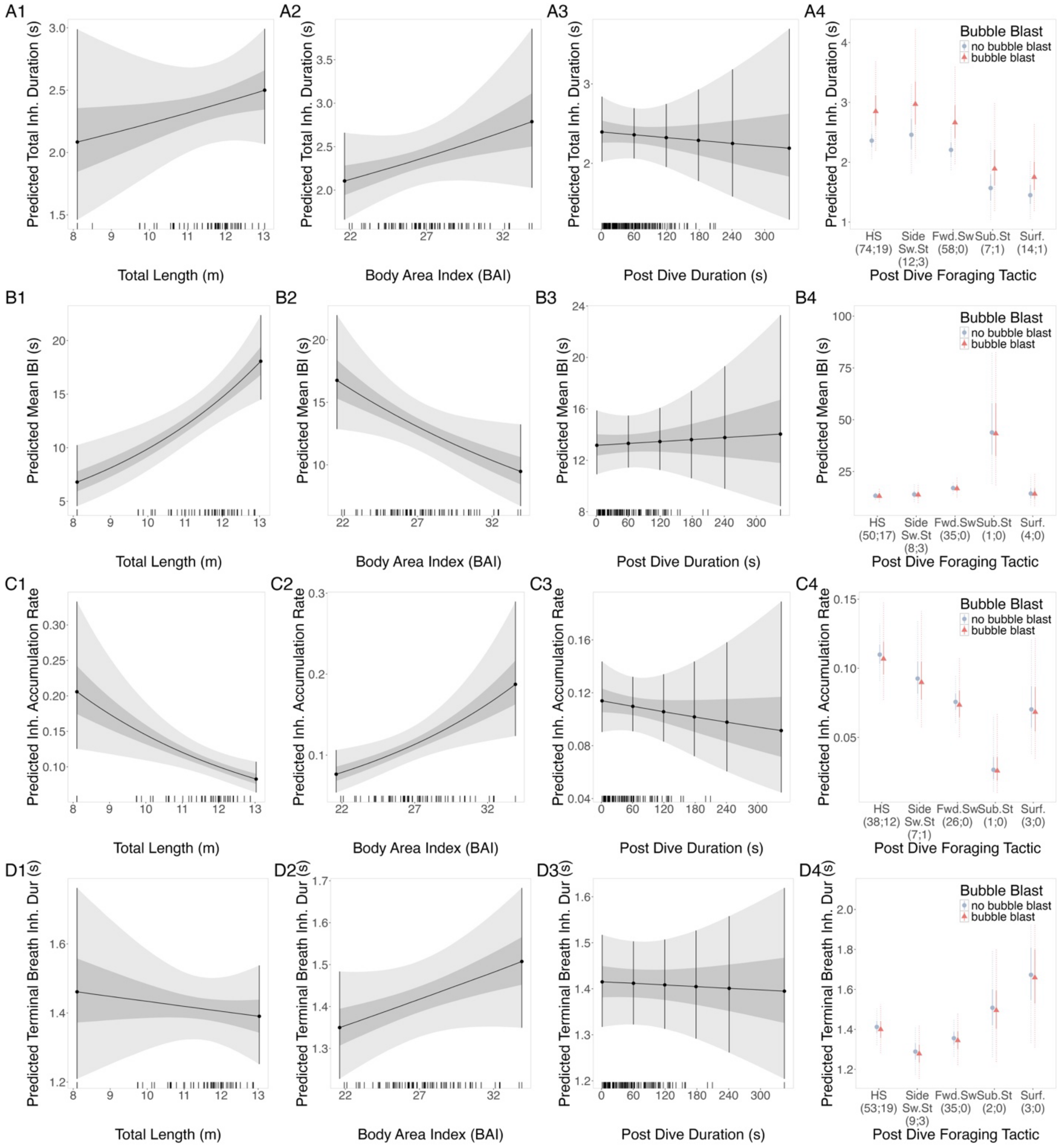

Figure S9. Estimated relationships from the "anticipation models" between (1) total length (TL), (2) Body Area Index (BAI), (3) proceeding dive duration (s) and (4) proceeding dive foraging tactic and bubble blast occurrence on (A) total inhalation duration (s), (B) mean inter-breath interval (IBI), (C) inhalation accumulation rate, and (D) inhalation duration of the terminal breath. All response variables were transformed from the log scale to seconds. In columns 1, 2, and 3, the line represents the mean posterior relationship, the dark gray shaded region represents the 50% credible interval, and the light gray shaded region represents the 95% credible interval. Points with bars represent the mean value and 95% credible interval at the

minimum and maximum (1) TL and (2) BAI. In (3) preceding dive duration, points with bars represent the mean value and 95% credible interval at the minimum and maximum dive duration and 1-,2-,3-,4- minute dive durations. The rug of vertical black lines along the x-axis in each plot represents the original data values. In column 4, the points represent the posterior mean values, the solid lines represent the 50% credible intervals, and the dashed lines represent the 95% credible intervals. In column 4 the tactics have been abbreviated as follows: HS = Headstand, Side.Sw.St = Side-swim stationary, Fwd.Sw. = Forward swimming tactics, Sub.St = Subsurface stationary, Surf. = Surface tactics. Grey circles indicate that no bubble blast occurred, while orange triangles indicated the occurrence of a bubble blast. The sample sizes per tactic are reported in parentheses under the tactic name: the first value indicates the number of observations with no bubble blast for that tactic, and the second value indicates the number of observations with a bubble blast for that tactic. The y-axis is fixed within each response variable.

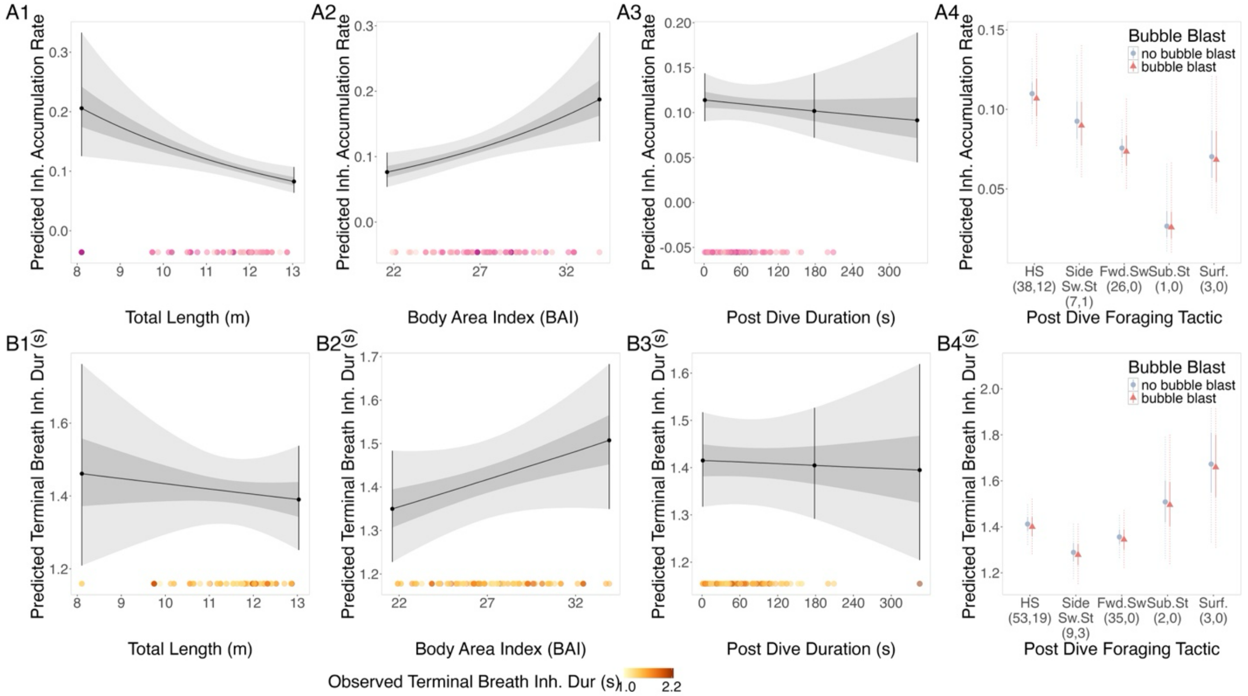

Figure S10. Estimated relationships from the "anticipation models" between (1) total length (TL), (2) Body Area Index (BAI), (3) following dive duration (s) and (4) following dive foraging tactic and bubble blast occurrence on (A) inhalation accumulation rate, and (B) inhalation duration of the terminal breath. All response variables were transformed from the log scale to seconds. In columns 1, 2, and 3, the line represents the mean posterior relationship, the dark gray shaded region represents the 50% credible interval, and the light gray shaded region represents the 95% credible interval. Points with bars represent the mean value and 95% credible interval at the minimum and maximum (1) TL and (2) BAI. In (3) preceding dive duration, points with bars represent the mean value and 95% credible interval at the minimum and maximum dive duration and 3-minute dive durations. The points along the x-axis in each plot represents the original data values, colored by the observed values of each row's respective response variable with darker shades representing higher values. In column 4, the points represent the posterior mean values, the solid lines represent the 50% credible intervals, and the dashed lines represent the 95% credible intervals. In column 4 the tactics have been abbreviated

as follows: HS = Headstand, Side.Sw.St = Side-swim stationary, Fwd.Sw. = Forward swimming tactics, Sub.St = Subsurface stationary, Surf. = Surface tactics. Grey circles indicate that no bubble blast occurred, while orange triangles indicated the occurrence of a bubble blast. The sample sizes per tactic are reported in parentheses under the tactic name: the first value indicates the number of observations with no bubble blast for that tactic, and the second value indicates the number of observations with a bubble blast for that tactic.

Table S11. Priors for all four anticipation models. All dive variables pertain to the dive proceeding the surface series.

name	coefficient	prior
Intercept	a	normal(0,1)
TL	bT	normal(0,1)
BAI	bBAI	normal(0,1)
Bubble blast occurrence	bBB_post	normal(0,1)
Proceeding dive duration	bDD_post	normal(0,1)
Forward swim tactics	bSWF_post	normal(0,1)
Subsurface stationary	bSBS_post	normal(0,1)
Side-swim stationary	bSSS_post	normal(0,1)
Surface/skim feeding	bSUF_post	normal(0,1)
Individual level random effect	sigma_id	uniform(0,10)

Table S12. Posterior predictive check results for all four anticipation models.

Model	Posterior p-value for mean(y)	Posterior p-value for sd(y)
Total Inhalation Duration	0.492	0.709
Inter-breath Interval (IBI)	0.475	0.672
Inhalation accumulation slope	0.531	0.674
Terminal breath inhalation duration	0.500	0.639

Recovery and Anticipation Model

Table S13. Posterior predictive check results for the recovery and anticipation model.

Model	Posterior p-value for mean(y)	Posterior p-value for sd(y)
Total Inhalation Duration	0.486	0.703

Table S14. Model coefficients for the recovery and anticipation model.

name	coefficient	prior	mean (95% CrI)
Intercept	a	normal(0,1)	1.01 (0.87,1.16)
TL	bT	normal(0,1)	0.01 (-0.11,0.13)
BAI	bBAI	normal(0,1)	0.05 (-0.08,0.17)
Preceding dive duration	bDD_pre	normal(0,1)	0.18 (0.08,0.28)
Proceeding dive duration	bDD_post	normal(0,1)	-0.05 (-0.15,0.06)
Forward swim tactics	bSWF_pp	normal(0,1)	-0.22 (-0.49,0.05)
Subsurface stationary	bSBS_pp	normal(0,1)	-0.67 (-1.27,-0.05)
Side-swim stationary	bSSS_pp	normal(0,1)	0.08 (-0.40,0.56)
Surface/skim feeding	bSUF_pp	normal(0,1)	-0.55 (-0.91,-0.19)
Individual level random effect	sigma_id	uniform(0,10)	0.12 (0.01,0.28)

Travel Model

Table S15. Summary of sample sizes, speeds, and respiration rates for the travel model run using UAS. While we did not model the tag data due to the inability to measure travel speed, we report the respiration rate for future reference.

Model	Data Source	No. travel series	No. Individuals	Travel Speed (m/s)	Respiration Rate (breaths/min)
Travel	UAS	40	29	1.63 (0.33)	1.59 (1.01)
	Tag	40	6	-	0.43 (0.3)

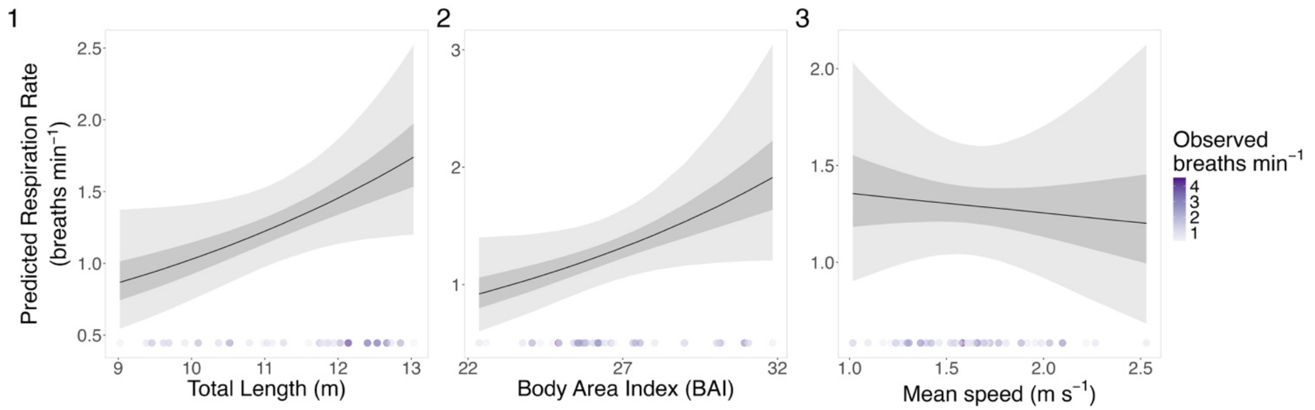

Figure S11. Estimated relationships during from the "travel model" between (1) total length (TL), (2) Body Area Index (BAI), (3) mean swim speed (m s^{-1}) on mean respiration rate from UAS data. The response variable was transformed from the log scale to respiration rate. The line represents the mean posterior relationship, the dark gray shaded region represents the 50% credible interval, and the light gray shaded region represents the 95% credible interval. The rug of vertical black lines along the x-axis in each plot represents the original data values.

Table S16. Posterior predictive check results for the travel model.

**Model**	**Posterior p-value for mean(y)**	**Posterior p-value for sd(y)**
Travel	0.495	0.689

Table S17. Model coefficients for the travel model.

			**Respiration Rate (UAS)**
**name**	**coeff**	**prior**	**mean (95% CrI)**
Intercept	a	normal(0,1)	0.27 (0.04,0.49)
TL	bT	normal(0,1)	0.11 (-0.12,0.34)
BAI	bBAI	normal(0,1)	0.08 (-0.13,0.30)
Speed	bS	normal(0,1)	-0.06 (-0.25,0.14)
Individual level random effect	sigma_id	uniform(0,10)	0.35 (0.06,0.66)

Exploratory Plots

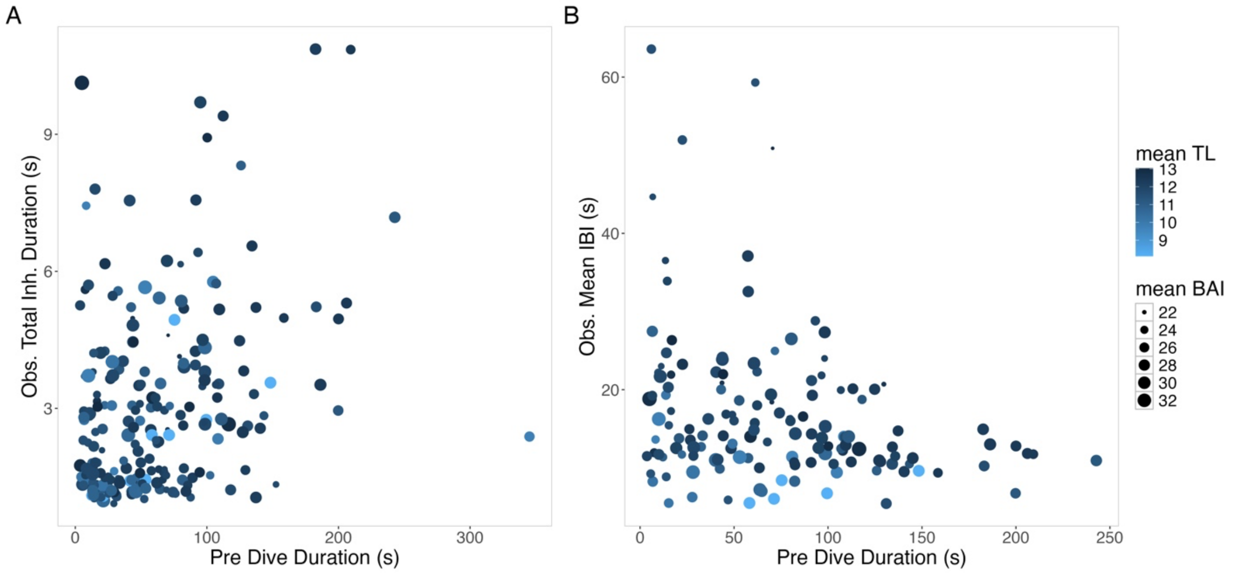

Figure S12. Relationship between preceding dive duration and observed (A) total inhalation duration and (B) mean inter-breath interval (IBI) with points colored by mean individual total length (TL) and sized by body area index (BAI).

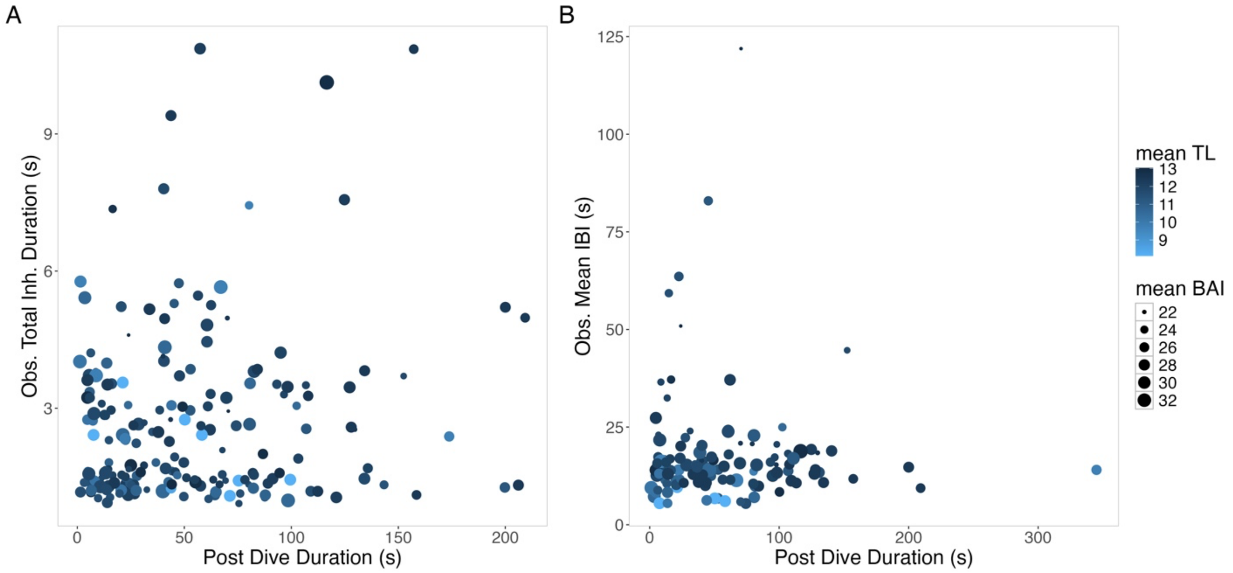

Figure S13. Relationship between proceeding dive duration and observed (A) total inhalation duration and (B) mean inter-breath interval (IBI) with points colored by mean individual total length (TL) and sized by body area index (BAI).

References

Bierlich, K.C., Wengrove, D., Bird, C.N., Davidson, R., Chandler, T., Torres, L.G., Cantor, M., 2024. LidarBoX: a 3D-printed, open-source altimeter system to improve photogrammetric accuracy for off-the-shelf drones. Drone Syst. Appl. 12, 1–10. https://doi.org/10.1139/dsa-2023-0051

Bird, C.N., Pirotta, E., New, L., Bierlich, K.C., Donnelly, M., Hildebrand, L., Fernandez Ajó, A., Torres, L.G., 2024. Growing into it: evidence of an ontogenetic shift in grey whale use of foraging tactics. Animal Behaviour 214, 121–135. https://doi.org/10.1016/j.anbehav.2024.06.004

Dawson, S.M., Bowman, M.H., Leunissen, E., Sirguey, P., 2017. Inexpensive Aerial Photogrammetry for Studies of Whales and Large Marine Animals. Frontiers in Marine Science 4. https://doi.org/10.3389/fmars.2017.00366
